# Urban population size and road traffic collisions in Europe

**DOI:** 10.1371/journal.pone.0256485

**Published:** 2021-08-27

**Authors:** Carmen Cabrera-Arnau, Steven R. Bishop

**Affiliations:** Department of Mathematics, University College London, London, United Kingdom; Universitat de Valencia, SPAIN

## Abstract

Millions of road traffic collisions take place every year, leading to significant knock-on effects. Many of these traffic collisions take place in urban areas, where traffic levels can be elevated. Yet, little is known about the extent to which urban population size impacts road traffic collision rates. Here, we use urban scaling models to analyse geographic and road traffic collision data from over 300 European urban areas in order to study this issue. Our results show that there is no significant change in the number of road traffic collisions per person for urban areas of different sizes. However, we find individual urban locations with traffic collision rates which are remarkably high. These findings have the potential to inform policies for the allocation of resources to prevent road traffic collisions across the different cities.

## Introduction

At a worldwide level, approximately every 24 seconds someone dies as a consequence of a road traffic collision [1]. For people aged 5 to 55, road traffic collisions are among the ten most common causes of death [2]. Besides the enormous emotional burden that each of these deaths leaves behind, they also lead to significant financial losses. For example, in Great Britain it is estimated that the average cost of in the year 2019 is above £100k ($140k), although for fatal traffic collisions, this figure could be as high as £2.2M ($3M) [3]. Much like wealth, road traffic collisions are not uniformly distributed across regions. At a global scale, road traffic collision death rates in low- and middle-income countries are about twice as large as in high-income countries (21.5 and 19.5 vs 10.9 per 100,000 population) [1]. At a national scale, road traffic collision fatality rates are higher in rural areas, but most traffic collisions actually take place in urban areas [4, 5].

Concurrently, the world is undergoing a rapid urbanisation process: it is estimated that by 2050, more than two thirds of the global population will live in urban areas, with the figure reaching 74% in Europe as of 2018 [6]. Given that most road traffic collisions take place in urban areas and the population size of these urban areas is likely to increase due to urbanisation, the following question arises: do road traffic collision rates increase with the population size of the urban area where they take place? As shown in [7], traffic congestion increases in urban areas of larger population size and more traffic congestion leads to more opportunities for collision. Additionally, traffic congestion can increase stress levels in drivers [8, 9], and this can also lead to a greater risk of collision [10, 11]. Due to these factors, an affirmative answer to the question above could be postulated. However, previous research on the issue of whether traffic congestion has an impact on road traffic collision rates has reached conclusions that might seem counterintuitive. For example, Shefer demonstrates, in a hypothetical situation, that a reduction in the level of congestion could inadvertently cause an increase in road fatalities [12]. However, Shefer only considers fatal collisions. Other works consider the total number of traffic collisions and reach different results. For instance, the authors in [13] analyse all the traffic collisions recorded by Shanghai Expressway Surveillance System in a three-year period and find that traffic exposure, congestion and merging behaviors all increase the risk of collisions on urban expressways. They also find that the risk factors are different in congested and non-congested flows. Despite the wealth of works on the topic, our current understanding of how traffic congestion affects collision risks is still limited. As it has been reported in the review by Retallack and Ostendorf [14], the dominant result in the literature is a positive linear relation between traffic collision rates and levels of congestion/traffic volume. But Retallack and Ostendrof also mention some works that analyse finer temporal resolution traffic data and show a U-shaped relationship.

Our aim here is to analyse the direct impact of urban population size on the incidence of road traffic collisions. We base our analysis on data from England and Wales, France, Germany and Spain. In order to achieve our aim, we firstly need to determine the population and number of road traffic collisions corresponding to the urban areas under consideration. However, there is no single way of establishing the boundaries of urban areas [15, 16] and different criteria are often chosen according to the type of analysis to be performed. Similar to the approach taken in [16], a classification based on commuting flows is used in this paper. Based on these classification criteria, cities and towns that have traditionally been considered as different entities, may be classified as the same urban area.

## Methods

In this work we present the results from processing geographic and road traffic collision microdata from England and Wales, mainland France, Germany and mainland Spain. England and Wales are two countries but, for ease of notation, we will refer to them as only one entity denoted by E&W. Similarly, mainland France, Germany and mainland Spain will be simply referred to as the countries France, Germany and Spain.

### Distribution of urban population sizes

In the Results section, we discuss the behaviour of road traffic collision rates in two types of urban areas from each of the four countries of interest: the largest urban areas and the rest of smaller urban areas. Hence, we consider it is necessary to give here a brief overview about the patterns displayed by the distribution of population sizes corresponding to the urban areas in E&W, France, Germany and Spain.

Urban population sizes have been found to follow heavy-tailed distributions, such as a power-law distribution [17–19] or a lognormal [20]. However, in practice, the population size of the largest urban area in a country is often larger than predicted by the underlying heavy-tailed distribution. These extremely large urban areas then become meaningful outliers and are sometimes referred to as dragon-kings, a term coined by Lahèrre and Sornette in [21]. Additionally, they have a special socioeconomic status forged by amplifying mechanisms for their own growth.

London and Paris would be examples of urban areas displaying dragon-king features. Their size is several times larger than the next largest urban area in their respective country and they are also primary nodes in the global socioeconomic network. Germany and Spain, however, are countries which have experienced a higher degree of territorial divide throughout history and where different cities have been appointed as the capitals at different periods in time. As a result, these countries have more than one urban area with an unexpectedly large population size and with a central role in the socioeconomic landscape of the country. In Germany, there are actually many urban areas that fulfil these characteristics, in particular, the ‘Big Five’ metropolitan regions (Berlin, Hamburg, the Rhine-Ruhr metropolitan region, Frankfurt and Munich); in Spain, Madrid and Barcelona.

### Population and number of road traffic collisions in the urban areas

The urban areas used here are the functional urban areas (FUAs) established by Eurostat [22], which are based on commuting flows [23]. The data corresponding to E&W, France and Germany is from 2018 and, in the case of Spain, from 2015.

The data corresponding to the small geographical hierarchies of each country is aggregated into urban areas and analysed further to produce the figures in the forthcoming sections. In the case of France, Germany and Spain, both population and road traffic collision data is collected by the local administrative unit (LAU). LAUs have different names in different countries: *communes* in France, *gemeinden* in Germany and *municipios* in Spain. In E&W, data is available for lower level geographic hierarchies known as Lower Layer Super Output Areas (LSOAs), designed specifically to improve the reporting of small area statistics. However, urban areas may extend over several of these small geographic hierarchies. For example, the urban area corresponding to Greater London would comprise 6,908 LSOAs while the urban area corresponding to Madrid would comprise 182 *municipios*.

Information related to population [24–27] as well as the shapefiles for the LSOAs [28], the LAUs and the FUAs [22] are publicly available for download. In the case of E&W, France and Spain, we downloaded data bases where each entry is a recorded road traffic collision [29–31]. For each traffic collision, the LSOA or LAU where it took place is specified. In the case of Germany, we used a data base where the traffic collisions are already aggregated by the LAU [32].

It is possible to make country-to-country comparisons of patterns that emerge as a result of considering a country’s urban system as a whole. However, a word of caution needs to be said about the comparability of population data corresponding to urban areas from different countries. The population in an urban area is computed as the sum of populations corresponding to the small geographical hierarchies that lie within the boundary of the urban area. However, these geographical hierarchies are country-dependent and, except in the case of the LSOAs in E&W, are also subject to historical agreements.

**Fig 1:**
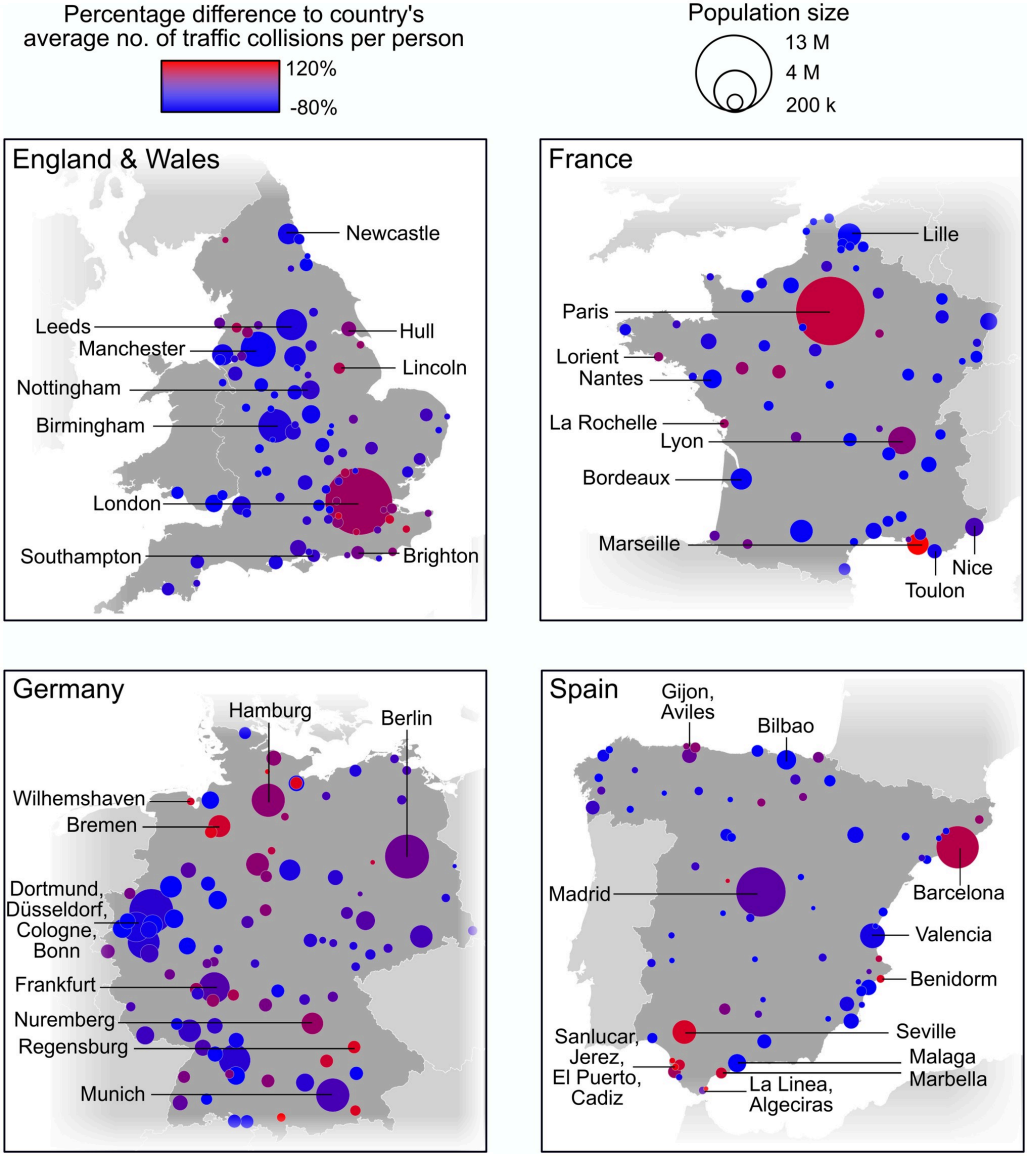
Map representation of the number of road traffic collisions per person in urban areas from England and Wales, France, Germany and Spain.

**Fig 2:**
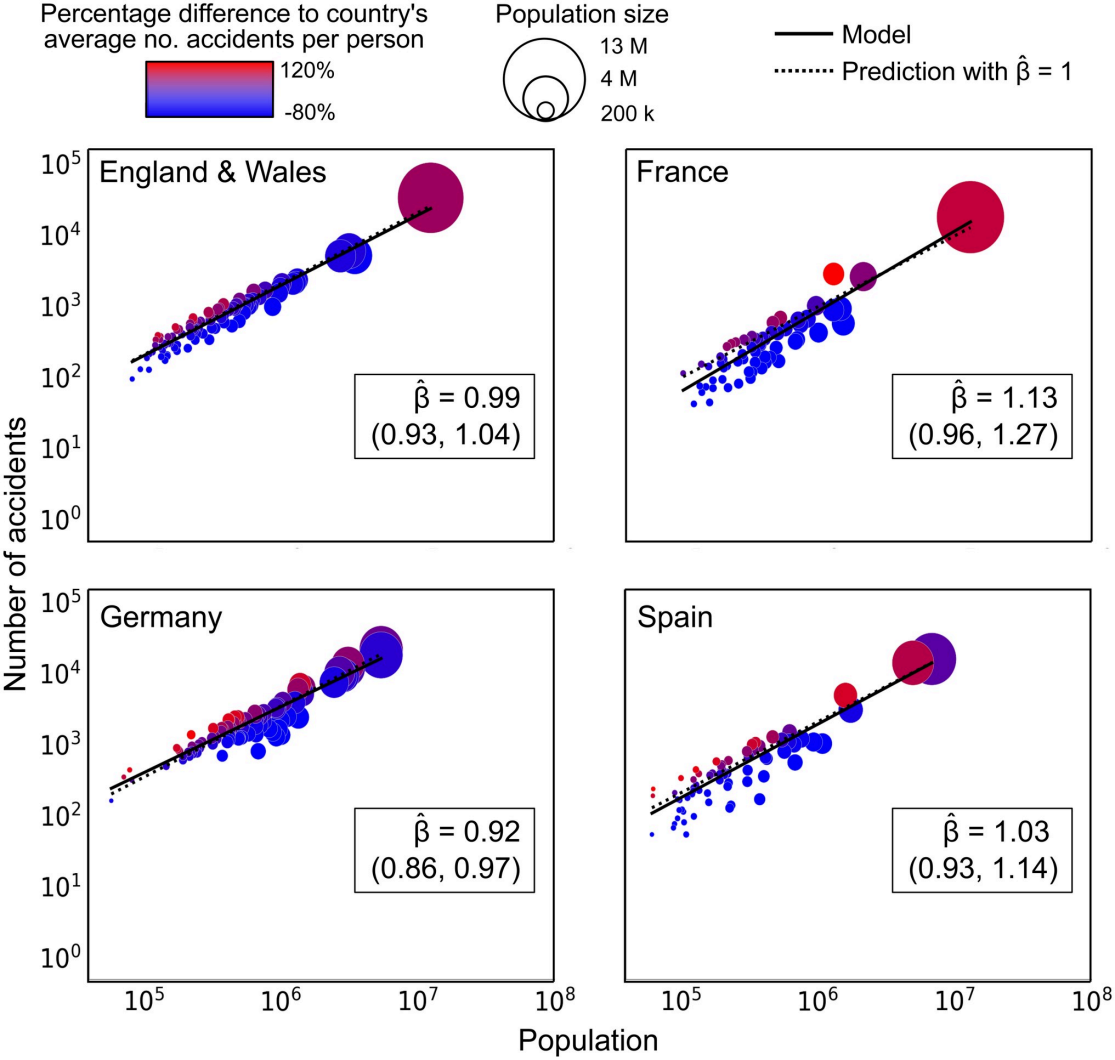
Urban scaling models corresponding to England and Wales, France, Germany and Spain.

Similarly, for all the countries under consideration, the number of traffic collisions in an urban area is obtained as the sum of the number of traffic collisions in each small geographical hierarchy that lies within the urban area’s boundary. But definitions as to what constitutes a traffic collision may also vary. For example, the traffic collisions recorded in France are those that required some form of medical treatment [30], whereas in E&W, all reported traffic collisions incurring personal injury, but not necessarily requiring medical care, are included in the national data base [33]. Furthermore, every country has different levels of under-reporting of data, especially when it comes to non-fatal traffic collisions. Data related to hospitalisation as a result of a traffic collision, surveys (e.g. National Transport Survey in Great Britain) and insurance compensation claims all indicate a higher number of casualties than are reported [33]. Hence, 300 traffic collisions per 100,000 people in an urban area from E&W does not quite mean the same as in a French, German or Spanish urban area. It is for this reason that in Figs 1 and 2, the colour key is based on the percentage difference between the number of traffic collisions per person in a given urban area and the corresponding country’s average number of traffic collisions per person in the urban areas under consideration.

We include as Supplementary Information the data sets that, upon processing of the raw data, have been used to generate Figs 1 and 2.

### Urban scaling models

Since Smeed’s 1949 pioneering work regarding statistical aspects of road traffic collisions [34], the precision and availability of both geographic and road safety data have improved considerably, enabling many other authors to expand the field [35–42]. Additionally, the more recent introduction of scaling models in the context of urban science [43] offers a new avenue for modelling road traffic collisions and understanding their behaviour. Urban scaling models are based on the hypothesis that a quantifiable property *Y* varies with city population size *X* according to

$$\begin{array}{c} Y\left(X\right)=\alpha X^{\beta } \end{array}$$ (1)

with scaling parameters *α* and *β*. According to the value of the scaling exponent *β*, the scaling model can display three types of behaviour. If 0 < *β* < 1, *Y* is said to grow sublinearly with *X*. Sublinear behaviour implies that the value of *Y* per person decreases with city population size. If *β* = 1, the scaling is linear and the values of *Y* per person are constant across city population sizes. If *β* > 1, *Y* scales superlinearly. When that is the case, the values of *Y* per person increase with city population size. Scaling models have been applied widely (see e.g. [44, 45]), in particular, we have previously used urban scaling models to describe the relationship between the number of traffic collisions of different degrees of severity and the population size corresponding to the set of ‘built-up’ areas (defined by a land-use classification criterion) from England and Wales [5]. In this paper, we extend the analysis to data from France, Germany and Spain as well as England and Wales.

An advantage of using urban scaling models is that they allow us to summarise the relative performance of cities across a vast range of population sizes under the same mathematical model. However, certain urban areas (frequently the largest ones in a region) are unique in that they play central roles in economic productivity of firms and workers [46], are especially prolific in certain industry sectors or have an extraordinary cultural output [47]. For this reason, it has been questioned [16] whether these urban areas, sometimes referred to as dragon-kings, should be analysed alongside the rest or whether on the contrary, they should be considered as a separate category. Here, we opt to include these urban areas in the analysis. Following the results in [48], we use a negative binomial regression for parameter estimation since it places less weight on larger urban areas, hence making the parameter estimation procedure more robust with respect to observations associated with large urban areas. Further, we test the performance of the negative binomial regression against a Poisson regression through the Akaike Infromation Criterion (AIC). For each country, we obtain that the former method yields a lower value of AIC, indicating that the negative binomial regression is a model of higher quality than the Poisson regression for the data sets of interest.

### Is the scaling behaviour significantly different from linear?

If the scaling behaviour was non-linear, the value of the estimated scaling exponent would have to be significantly different from 1. In order to test for significance, we perform a Monte Carlo simulation. Let us assume the null hypothesis that the data corresponding to country *A* comes from a scaling model with parameter *β*_0_ = 1. We also estimate the parameters of the original sample, which we denote as ${\hat{\alpha }}_{A}$ and ${\hat{\beta }}_{A}$. In each iteration *i* of our simulation, we follow the steps below:

- We keep the populations of the urban areas from country *A* the same as in the original sample.
- Then, we generate random values for *Y* distributed according to a negative binomial distribution with mean $\mu ={\hat{\alpha }}_{A}X^{{\hat{\beta }}_{0}}$ and variance *σ*^2^ = *μ* + *μ*^2^.
- Finally, we estimate the value of the scaling exponent ${\hat{\beta }}_{A}^{i}$ corresponding to the sample generated in the *i*th iteration and store it.

Once we have completed the simulation process, we will have an estimated value of the scaling exponent for each iteration. Then we compute the *p*-value as the proportion of stored values which satisfy $|{\hat{\beta }}_{A}^{i}-\beta_{0}\left|>\right|{\hat{\beta }}_{A}-\beta_{0}|$. If the *p*-value is smaller than a chosen significance threshold of 0.05, our null hypothesis can be rejected.

Applying 2,000 iterations of this method to the four countries under consideration, we obtain the following *p*-values: *p*_*E*&*W*_ = 0.93, *p*_*FR*_ = 0.55, *p*_*DE*_ = 0.61 and also *p*_*ES*_ = 0.81 for E&W, France, Germany and Spain respectively, which are all above the chosen level of significance.

## Results

### Geographical distribution of road traffic collisions in urban areas

In Fig 1, we have plotted the population and number of traffic collisions per person corresponding to the urban areas from E&W, France, Germany and Spain. We have opted for a map layout, as this helps with visualisation and understanding.

#### Largest urban areas

We observe in Fig 1 that the largest urban areas in E&W and France (London and Paris) stand out in terms of their large population size and high number of traffic collisions per person. This is not the case for the German and Spanish counterparts, Berlin and Madrid. In Germany, the urban areas are more evenly spread across the whole range of population sizes, and so is the number of road traffic collisions. In Spain, there are two urban areas, instead of just one, that stand out for their population size: Madrid and Barcelona. The number of road traffic collisions is also relatively high. The fact that the four countries display different patterns is perhaps not so surprising, considering that their urban areas have been subject to unique historical developments. More details regarding this observation are provided in the Discussion and conclusions section.

#### Other urban areas

The number of road traffic collisions in smaller urban areas displays a high variability in all the countries. As a consequence, we cannot discern, *a priori*, whether urban population size plays a role in determining the number of road traffic collisions for these urban areas. The fact that there is such degree of variability is an indication that there might be variables other than urban population size which affect road traffic collision rates. For example, in E&W, Sheffield and Stevenage have similar road traffic collision rates of approximately 175 per 100,000 people, however, the functional urban area corresponding to Sheffield has a population size of 1.3 million, whereas the one corresponding to Stevenage has a population of just above 100,000 people. Similarly, Bremen and Willemshaven in Germany have a road traffic collision rate of around 500 per 100,000 people, but their population sizes are also very different: Bremen’s functional urban area has a population size of 1.4 million, whereas Willemshaven has a population size just below 175,000.

### Scaling of traffic collisions in urban areas

After inspecting individual cities in the previous section, a natural question arises as to whether the population size of a city has an effect on the number of traffic collisions per person. To answer this question, we firstly propose the scaling hypothesis, which assumes that the number of road traffic collisions *Y* in a given urban area is determined by its population size *X* according to an urban scaling model of the form *Y* = *αX*^*β*^. The two parameters associated with this model, *α* and *β*, can be estimated from the data. If the parameter *β*, known as scaling exponent, is found to be significantly larger than 1, then this gives an indication that the number of road traffic collisions per person in an urban area increases with population size. In order to estimate the scaling exponent, we take into account the considerations from [49] and [48], where the authors emphasise the need to account for the statistical properties of the data. Here, we do this by using a generalised linear model for regression. Details about this approach, the computation of confidence intervals for the parameters and more background about urban scaling models are provided in the Methods section.

Fig 2 shows the data related to the urban areas in the four countries of interest as well as the scaling model that provides the best fit to the data, with 95% confidence intervals obtained by bootstrapping. In E&W and Germany, the estimated scaling exponent $\hat{\beta }$ has been calculated to be slightly below 1, while in France and Spain, it is slightly above 1. However, in the methods section we show that *β* is not significantly different from one (*p* > 0.05) in all four countries, hence indicating that, for the definitions of urban areas and road traffic collisions used here, there are no significant effects of urban population size on road traffic collisions.

In Fig 2, the high variability in road traffic collision rates for urban areas of a given population size is perhaps even more evident. This is an indicator that, quite possibly, there are more variables influencing road traffic collision rates apart from urban population size. If this is the case, urban scaling models should be replaced for other models that incorporate these additional variables.

## Discussion and conclusions

We conclude that urban population size has no significant effect on the number of road traffic collisions in urban areas from four European countries. This conclusion is based on the results obtained through the application of urban scaling models, which uncover patterns that emerge at a country-wide level.

These findings are in contrast with our results in [5], where we applied urban scaling models to describe the relation between the number of road traffic collisions and the population of built-up areas from E&W (defined according to a land-use criterion). On that occasion, we found that the number of road traffic collisions scales superlinearly with urban population size. The discrepancies between the results are due to the fact that we considered different urban areas and a different regression method for the estimation of parameters.

We should point out here that the behaviour of large cities, sometimes called dragon-kings, is difficult to model due to their unique characteristics [16, 18]. Therefore, following results in [48], we chose a generalised linear model for the estimation of the scaling model parameters that accounts for the dragon-kings’ unpredictability. This is reflected through the fact that our choice of generalised linear model assumes wider probability distributions for the number of traffic collisions as the population size of the urban areas increases.

Turning the attention to individual urban areas, we observe cases where the road traffic collision rate is remarkably higher than the national average for a given population size. This tendency could be the result of the fact that other variables (volumes of traffic; traffic congestion; proximity to a port) may be playing a key role in determining the number of collisions, but they are not analysed in the paper.

In particular, we highlight two types of behaviour displayed by individual urban areas: that corresponding to the largest urban areas in a country and that corresponding to the rest of smaller urban areas. Firstly, we find that the top largest urban areas in E&W and France, London and Paris respectively, display high collision rates with respect to each country’s average. Both E&W and France are countries that, despite their different levels of centralisation [50], have remained relatively unified in recent history. This has allowed their capital cities to forge their pivotal role, not only at a national level, but also as global cities [51]. Here, we find that both urban areas corresponding to the capital cities are also special when it comes to their number of road traffic collisions per person. In contrast, Germany and Spain have either only been unified recently or have experienced surges of internal divide [52]. As a consequence, they display several urban areas that compete somewhat for the leadership. In Germany, the ‘Big Five’ metropolitan regions (Berlin, Hamburg, the Rhine-Ruhr metropolitan region, Frankfurt and Munich) are all prominent in terms of investment and market development. In Spain, there are two main urban areas with a central socioeconomic role: Madrid and Barcelona. Analogously, the incidence of road traffic collisions is more spread across all of these urban areas instead of concentrating in just one as it was the case for E&W and France.

Secondly, the number of road traffic collisions in smaller urban areas displays high variability in all the countries. This effect could be attributed to the different volumes of traffic found in different locations, since traffic flow [53] and, arguably, traffic congestion [5], have been shown to be positively correlated with traffic collision rate. For example, in E&W, our results recognise Hull and Lincoln as traffic collision hotspots, which have also been ranked among the top ten cities with the highest levels of traffic congestion in the UK [54]. We also detect a remarkable concentration of urban areas with an above-average incidence of road traffic collisions per person on the South East of England, including London’s satellite urban areas and other coastal urban areas. Like Hull, some of these locations are near to large ports. Ports are freight-generating points and hence, attract heavy goods vehicles from other places. Depending on how accommodating the surrounding infrastructure is, ports can therefore limit the urban space, while at the same time, increasing traffic flow [55] and leading to more road traffic collisions. Other urban areas that have a sea or river port are La Rochelle, Loirent and Marseille in France; Bremen, Hamburg and Regensburg (Danube port) in Germany and Aviles, Cadiz, Gijon and Seville (Guadalquivir port) in Spain. The number of road traffic collisions in all of these locations is above the country’s average.

Our results show how road traffic collisions are spread across the different urban areas of four countries and therefore, can help determine the top-priority regions to be targeted by policies for the alleviation of disruption caused by road traffic collisions. Particularly, our findings should be considered when countries apply any levelling-up strategies to improve aspects of certain regions that are yet to reach the overall national standard.

It remains as future work to improve our understanding of the causes that lead to the unusually elevated number of traffic collisions in certain urban areas. This can be done by studying, for example, the traffic flow levels in these urban areas or their particular demographic composition, since it has been shown that certain demographic groups have an increased risk of being involved in road traffic collisions [56]. Other directions for future research include an analysis based on choices of urban areas other than the Eurostat functional urban areas or restricted to only a type of traffic collision, e.g. fatal traffic collisions.

This leads us to mention some major limitations that we have encountered when performing this research. While Eurostat attempts to unify the urban areas for different countries, the definitions are still county-dependent, since they are based on the particular geographical hierarchies established by each country. Hence, the comparability between the results corresponding to the countries analysed here is compromised. Furthermore, the definitions provided by Eurostat are obviously limited to European countries. In this context, we remark the need to standardise road safety data definitions and collection procedures so that more low-income countries, which tend to be the most affected by road traffic collisions, can be included in the body of research, in line with the central, tranformative promise of the 2030 Agenda for Sustainable Development and its Sustainable Development Goals (SDGs) [57] to ‘Leave No One behind’.

## Supporting information

S1 Table Population and number of road traffic collisions in functional urban areas from England & Wales. (CSV)

S2 Table Population and number of road traffic collisions in functional urban areas from France. (CSV)

S3 Table Population and number of road traffic collisions in functional urban areas from Germany. (CSV)

S4 Table Population and number of road traffic collisions in functional urban areas from Spain. (CSV)
